# The American Medical Almanac for 1848

**Published:** 1847-12

**Authors:** 


### ARTICLE X.

*The American Medical Almanac for* 1848. Containing statistics of the various Medical Colleges, Hospitals, Dispensaries, etc., of the United States, together with other information of value to the physician and student. Philadelphia: Lindsay & Blakiston. pp. 224, 18mo. (From the Publishers.)

This is a neat and convenient little volume, that furnishes an almanac convenient for office use, and at the same time furnishes an amount of statistical information in reference to medical institutions, that must be interesting to the physician, not to be found collated elsewhere. We hope the enterprising publishers may meet with sufficient encouragement to induce them to continue its publication annually hereafter. E.

